# Membrane Fusion Proteins of Type I Secretion System and Tripartite Efflux Pumps Share a Binding Motif for TolC in Gram-Negative Bacteria

**DOI:** 10.1371/journal.pone.0040460

**Published:** 2012-07-06

**Authors:** Minho Lee, So-Young Jun, Bo-Young Yoon, Saemee Song, Kangseok Lee, Nam-Chul Ha

**Affiliations:** 1 Department of Life Science, Chung-Ang University, Seoul, Republic of Korea; 2 Department of Manufacturing Pharmacy, College of Pharmacy, Research Institute for Drug Development, Pusan National University, Busan, Republic of Korea; Centre National de la Recherche Scientifique, France

## Abstract

The Hly translocator complex of *Escherichia coli* catalyzes type I secretion of the toxin hemolysin A (HlyA). In this complex, HlyB is an inner membrane ABC (ATP Binding Cassette)-type transporter, TolC is an outer membrane channel protein, and HlyD is a periplasmic adaptor anchored in the inner membrane that bridges HlyB to TolC. This tripartite organization is reminiscent of that of drug efflux systems such as AcrA-AcrB-TolC and MacA-MacB-TolC of *E. coli*. We have previously shown the crucial role of conserved residues located at the hairpin tip region of AcrA and MacA adaptors during assembly of their cognate systems. In this study, we investigated the role of the putative tip region of HlyD using HlyD mutants with single amino acid substitutions at the conserved positions. *In vivo* and *in vitro* data show that all mutations abolished HlyD binding to TolC and resulted in the absence of HlyA secretion. Together, our results suggest that, similarly to AcrA and MacA, HlyD interacts with TolC in a tip-to-tip manner. A general model in which these conserved interactions induce opening of TolC during drug efflux and type I secretion is discussed.

## Introduction

Gram-negative bacteria have developed sophisticated secretion systems due to their characteristic double-layer membranes. Secretion of the cytotoxin α-hemolysin (HlyA) in many uropathogenic strains of *Escherichia coli* is mediated by the protein complex Hly translocator, which belongs to the type I secretion system [1]. The 108-kDa HlyA can penetrate the membrane of host cells, creating a pore and causing them to lyse, which is an essential step for the bacteria to begin the infectious process [2].

The Hly translocator is comprised of HlyB, HlyD, and TolC [3]. The inner membrane protein HlyB is a member of the ATP-Binding Cassette (ABC) transporter family and transports HlyA from the cytosol by utilizing ATP hydrolysis [4], [5]. TolC is the outer membrane channel protein and is often the final portal in the transport pathways of protein toxins or toxic molecules that have entered the cell [6], [7], [8]. HlyD is an adaptor protein that links HlyB to TolC, providing a continuous transmembrane duct for the export of HlyA. Previous studies have shown that HlyB and HlyD form a stable translocator complex *in vivo* in the absence of either HlyA or TolC, and TolC is then recruited into the translocator in the presence of the substrate HlyA [9]. A similar organization has been implicated in tripartite efflux pumps [10], [11].

HlyD has a small N-terminal cytosolic domain connected to a large periplasmic domain by a single transmembrane helix [12]. It has been proposed that adaptor proteins share a similar overall structural organization and conserved regions only in their C-terminal periplasmic domain [10]. The N-terminal cytosolic domain is unique in HlyD, and the transmembrane region is lacking or substituted with a lipid anchor in some of adaptor proteins [10]. Structural data for several functional homologues such as *E. coli* AcrA, MacA and CusB and *Pseudomonas aeruginosa* MexA that contribute to drug or metal efflux systems have shown theC-terminal periplasmic domain [13], [14], [15], [16]. The periplasmic domains of AcrA, MacA, CusB, and MexA commonly comprise a membrane proximal (MP) domain, a β-barrel domain, a lipoyl domain, and an α-helical domain that are linearly arranged in the tertiary structures [13], [14], [15], [17], [18], [19]. Although all the α-helical domains are responsible for binding to their cognate outer membrane channel protein [20], [21], [22], [23], they are structurally variable. The α-helical domains of AcrA and MacA consist of a long α-hairpin of different lengths [13], [14], while that of CusB is folded into a three-helix bundle structure [15], [16]. Crystal structures, combined with genetic studies, have established that MacA and CusB exhibit a funnel-like hexameric assembly in their functional states [14], [21], [22], [24].

AcrA and MacA are functional and structural homologues of HlyD, and these are commonly associated with TolC in *E. coli.* However, HlyD has a relatively low sequence similarity with AcrA and MacA compared with the homology between AcrA and MacA. The α-helical domain of HlyD is not clearly defined by prediction programs unlike AcrA and MacA, suggesting that the TolC binding motif of HlyD might be different from that of the other adaptor proteins. Thus, the oligomeric state and TolC binding model of HlyD remains ambiguous.

Our research group has investigated the assembly of drug efflux pumps and proposed a tip-to-tip binding model between MacA (or AcrA) and TolC [14], [20], [21], [22], [23], [25]. According to the model, the α-helical tip region of the adaptor protein makes a cogwheel-to-cogwheel interaction with the TolC α-barrel tip region. In this study, we provide experimental evidence indicating that HlyD shares this mechanism of TolC binding.

## Results

### Sequence analysis of the putative HlyD α-helical region

Since HlyD, MacA, and AcrA proteins are commonly associated with TolC, we hypothesized that HlyD shares a common structural motif with MacA and AcrA for binding to TolC. It should be noted that the conserved RxxxLxxxxxxS motif (x stands for any residue; we designate it the RLS motif), located at the α-hairpin tip region of MacA, AcrA, and MexA, creates the binding interface for the TolC α-barrel tip [21], [22], [23]. The RLS motifs are universally located at the cogwheel region of the funnel-like hexamers of MacA, AcrA, and MexA [14], [22], [23], [25]. Single mutations at the conserved arginine, leucine, and serine residues within the RLS motif affect the activity of efflux adaptors (i.e., direct binding to TolC *in vitro* and drug efflux *in vivo*) [20], [21]. Here, it is worth noting that the S143A substitution in MacA retains some activity while the S143Y does not. This could be due to the bulkiness of tyrosine but also suggests that the serine residue of the RLS motif is more permissive to mutations (or less important for protein activity) [21].

**Figure 1:**
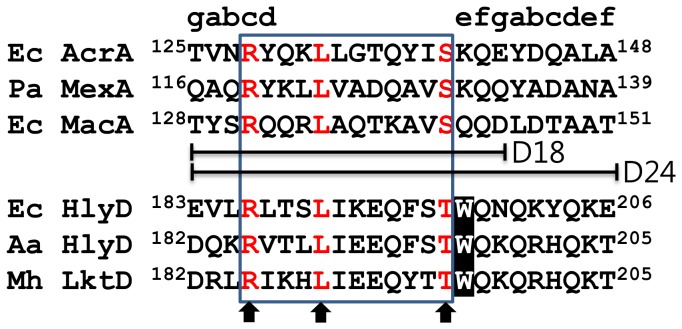
Sequence comparison of the α-hairpins with the anti-parallel coiled-coil structures. Sequences of the RLS (or RLT) motif from six adaptor proteins are aligned. The corresponding heptad positions are shown above the sequence. The three conserved residues in the RLS (or RLT) motif are indicated by arrows. The12 RLS (or RLT) motif residues are in a box, which was used in the MacA-HlyD12 hybrid. The 18 and 24 residues used in the construction of MacA-HlyD18 and MacA-HlyD24, respectively, are indicated (D18 and D24). The amino acid numbering is based on the protein precursors. In the sequence alignment, Ec, Pa, Aa, and Mh stand for *E. coli*, *P. aeruginosa*, *Actinobacillus actinomycetemcomitans*, and *Mannheimia haemolytica*, respectively.

Since HlyD does not show a significant sequence similarity with MacA or AcrA, we did a manual sequence alignment by considering the spacing between the conserved residues of the RLS motif. A putative RLS motif was identified in the region encompassing amino acids 186 to 197 in *E. coli* HlyD. This motif was also found in a subset of HlyD proteins from other species and homologous adaptor proteins engaged in other type I secretion systems. In this motif, serine was changed to threonine with conservation of a hydroxyl-bearing amino acid (Fig. 1). The motif has a substitution at the third serine site with a threonine residue, and thus we designated the motif as the RLT motif. This substitution at the third conserved residue seems reasonable because functional flexibility was observed at the residue in some adaptor proteins, as mentioned above. Further sequence similarity was present within the RLT motif, indicating that this motif is functionally important among HlyDs (Fig. 1).

### Arg186, Leu190, and Thr197 of HlyD are important in the secretion of HlyA *in vivo*

**Figure 2:**
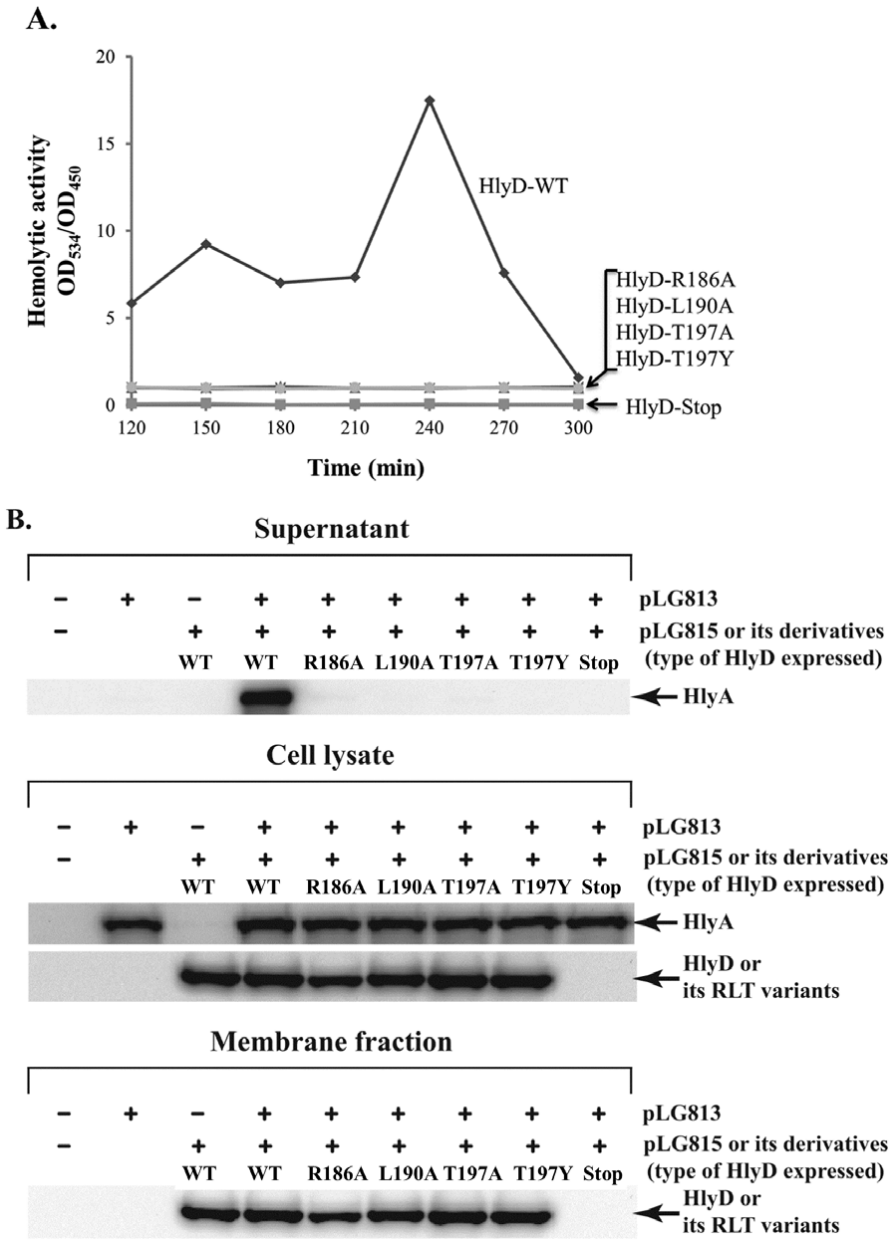
HlyA activity of *E. coli* SE5000 expressing wild-type or mutant HlyD. A. Hemolytic activity of *E. coli* cells expressing wild-type or mutant HlyD. The absorbance at 450 and 543 nm was detected using a spectrophotometer, and hemolytic activities were calculated according to the following formula: Percent Hemolysis = (1-ODs/ODt)×100, where ODs and ODt are the differences in optical density at 543 nm between the samples and 100% hemolyzed SRBC solution, and differences in optical density at 450 nm between nonhemolyzed (control) and 100% hemolyzed sheep red blood cell solution, respectively. Filled symbols and lines indicate hemolytic activity detected in culture supernatants (OD_543_/OD_450_). B. Effects of expression of HlyD mutants (HlyD-R131A, HlyD-L186A, HlyD-T190A, HlyD-T197Y, HlyD-null) on HlyA secretion. *E. coli* SE5000 coexpressing HlyA, HlyB, HlyC, and wild-type or HlyD mutants (HlyD-R186A, HlyD-L190A, HlyD-T197A, HlyD-T197Y, or HlyD-Stop) were grown exponentially in LB with 10 mM CaCl_2_ at 37°C, and proteins in the culture supernatant (equivalent to 3.5 OD_600_ of cells) were TCA precipitated. The precipitated protein samples were separated by SDS-PAGE and subjected to immunoblotting with HlyA antibody (upper panel). Total cell lysate was used for the western blot analysis of HlyA to detect its expression levels in the cell (lower panel).

To examine the functional importance of the three residues in the HlyD RLT motif, the residues were mutated in a cloned copy of *hlyD* in the plasmid pLG815, which directs the expression of *hlyB* and *hlyD* genes under the control of the *cat* gene promoter [26] (Table 1). In addition, the start codon of the *hlyD* gene in pLG815 was mutated to a stop codon, and the resulting plasmid (pLG815-HlyD-null) was used as a negative control for *hlyD* expression. The created pLG815-derived plasmids (pLG815-HlyD-null, pLG815-HlyD-R186A, pLG815-HlyD-L190A, pLG815-HlyD-T197A, and pLG815-HlyD-T197Y) were further modified by inserting a DNA segment encoding a c-Myc epitope (EQKLISEEDL) at the C-terminus of the HlyD coding region in pLG815, resulting in a pLG815-HlyD-c-Myc series of plasmids. The pLG815-HlyD-c-Myc series of plasmids were transformed into *E. coli* strain SE5000, harboring pLG813, which directs the expression of *hlyA* and *hlyC* under the control of their intact promoter (Kenny *et al.*, 1992). The resulting transformants were tested for their ability to hemolyze red blood cells. When HlyD mutants containing R186A, L190A, or T197Y were coexpressed with HlyA, HlyB, and HlyC in *E. coli* strain SE5000, the degree of hemolytic activity of *E. coli* cells on the M63-blood agar, as well as in liquid medium, were indistinguishable from those of *E. coli* cells expressing the *hlyD* null mutant (Table 1 and Fig. 2A). Expression of HlyD containing the T197A mutation in *E. coli* cells resulted in the formation of a clear zone on the M63-blood agar that was ten times smaller in diameter than that formed by *E. coli* cells expressing wild-type HlyD, indicating that this mutant rendered *E. coli* cells partially hemolytic (Table 1). However, *E. coli* cells expressing HlyD-T197A did not exhibit hemolytic activity that was detectable in the liquid medium, whereas those expressing wild-type HlyD showed strong hemolytic activity (Fig. 2A).

**Table 1:** Degree of hemolysis by *E. coli* cells expressing HlyD mutants.

HlyD mutation			Degree of hemolysis (diameter of a clear zone^1^)
HlyD mutant	Amino acid change	Codon change	
HlyD WT	None	None	2.5±0.25 cm
HlyD null	M1→Stop	ATG→TAG	None
HlyD R186A	R186→A	CGT→GCT	None
HlyD L190A	L190→A	TTG→GCT	None
HlyD T197A	T197→A	ACA→GCT	0.2±0.02 cm
HlyD T197Y	T197→Y	ACA→TAT	None

1 ∼10^4^ cells were spotted on M63-blood agar plate, incubated for 24 hrs, and the diameter of the clear zone was measured.

We further confirmed that the observed hemolytic activity of *E. coli* cells expressing HlyD mutants was related to the HlyA levels secreted through the Hly translocator complex. *E. coli* strain SE5000 coexpressing HlyA, HlyB, and HlyC together with wild-type or mutant derivatives of HlyD were grown in LB medium with 10 mM CaCl_2_ at 37°C for 4 hr to an OD_600_ of 3.5. These conditions induce maximal hemolysin secretion [26], [27]. Culture supernatants and cell pellets were separated by centrifugation. Proteins were separated by SDS-PAGE and analyzed by Western blot with antibodies directed against HlyA. As shown in Fig. 2B (middle), the same amounts of HlyA were detected in cell extracts expressing wild-type or mutant HlyD proteins. The wild-type and mutant HlyD proteins tagged with the c-Myc epitope were normally expressed in the cell and inserted into the membrane fraction, according to Western blot with anti-c-Myc antibodies (Fig. 2B middle and bottom). As expected, HlyA was secreted into the medium when cells expressed wild-type HlyD. In contrast, HlyA was very poorly or not secreted from cells expressing HlyD proteins with mutation in the RLT motif (Fig. 2B top). These results demonstrate that Arg186, Leu190, and Thr197 play an important role in the function of HlyD *in vivo* and further suggest the functional importance of the HlyD RLT motif in the function of the Hly translocator.

### Physical interaction between HlyD RLT motif and TolC *in vivo*

We have previously shown that the RLS motif of the tip region in MacA and AcrA is crucial for binding to the tip region of TolC. Therefore, we investigated the interaction between TolC and the RLT motif of HlyD using *in vivo* chemical cross-linking [9]. In this experiment, we used a pLG815-HlyD-c-Myc series of plasmids to express wild-type or mutant HlyD tagged with c-Myc at the C-terminus, and HlyB. pLG813 was used to express the substrate HlyA and HlyC. Plasmid pTolC2 has a replication origin from pSC101 [28] and an ampicillin antibiotic marker, which are compatible with pLG813- and pLG815-derived plasmids. The plasmid pTolC2 contained a cloned copy of the *tolC* gene, which directs the synthesis of TolC with a hexahistidine-tag at the C-terminus under the control of a *lacUV5* promoter. The three plasmids, pTolC2, pLG813, and one of the pLG815-HlyD-c-Myc series were transformed into an *acrAB*- and *tolC*-deleted *E. coli* strain. Transient protein complexes were stabilized with the chemical cross-linker DSP, which has a fixed 12-Å spacer arm that connects the primary amine groups of two adjacent proteins and contains a disulfide bond cleavable under reducing conditions. Cells were lysed and protein complexes were isolated by affinity chromatography via the His-tagged component (TolC-His). The eluted complexes were treated with sample buffer containing DTT and boiled to cleave the DSP molecule and release the individual components. These were resolved by SDS-PAGE and identified by immunoblotting using anti-His (TolC) and anti-c-Myc (HlyD variants) antibodies.

**Figure 3:**
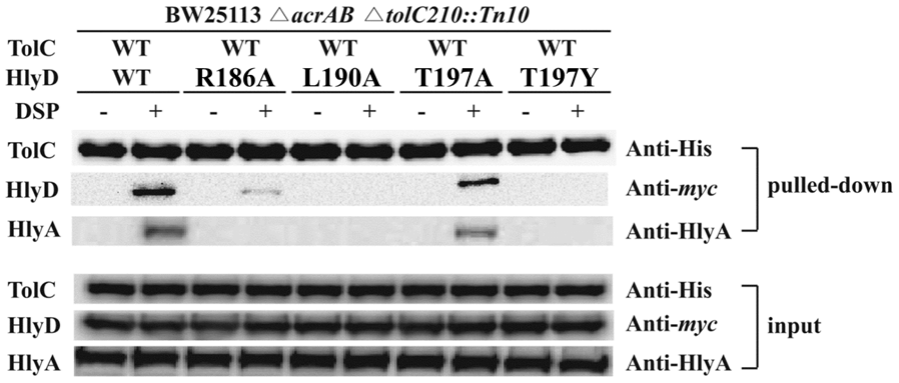
Interaction between TolC and HlyD *in vivo*. The *in vivo* interaction between HlyD and TolC was detected using a chemical cross-linking agent (DSP). *E. coli* BW25113Δ*acrAB* Δ*tolC210::Tn10* cultures that coexpressed HlyA, HlyB, HlyC, hexa-His-tagged wild-type TolC, and c-Myc-tagged wild-type HlyD (WT) or one of the HlyD mutants (R186A, L190A, T197A and T197Y) are shown. All cultures were treated with (+) or without (−) DSP. Affinity-purified TolC and cross-linked proteins (HlyA and HlyD) were separated by SDS-PAGE and immunoblotted using monoclonal antibodies to His-tag and c-Myc, and polyclonal antibodies to HlyA.

As shown in Fig. 3, the crosslinking of HlyD-R186A and HlyD-T197A to TolC were 10% and 48% of that of wild-type HlyD to TolC, respectively, whereas crosslinking of HlyD-L190A and Hly-T197Y to TolC was not detected. These results indicated that an amino acid substitution in the RLT motif partially (R186A and T197A) or completely (L190A and T197Y) abolished the binding of HlyD to TolC *in vivo*. These findings are consistent with the pivotal role of the RLT motif in the secretion of HlyA *in vivo*, even with the flexibility at the third residue. Moreover, the wild-type HlyD mediated the access of HlyA to TolC, detected by using the crosslinker, whereas the mutant HlyD abolished or reduced the accessibility of HlyA to TolC (Fig. 3). Taken together, our results strongly suggest that the RLT motif of HlyD makes direct contact with TolC, as is the case with the RLS motif of MacA or AcrA.

### The RLT motif plays an important role in binding to the TolC α-hairpin tip region *in vitro*

**Figure 4:**
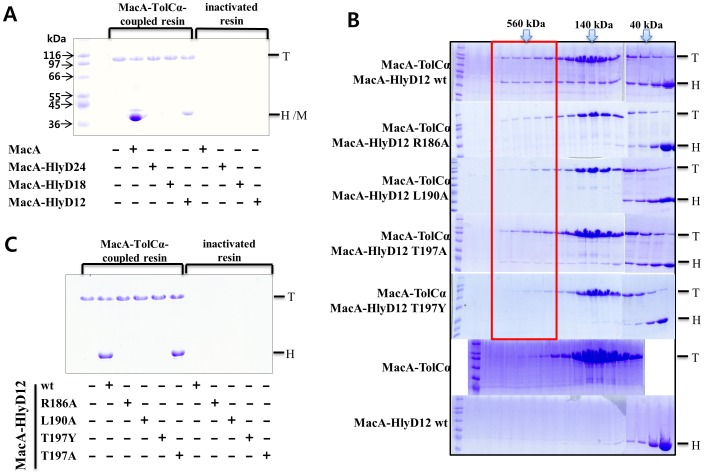
Interaction between the HlyD RLT motif and the TolC α-barrel tip region. A. The MacA-TolCα hybrid (T) was coupled to the CNBr-activated resin or the inactivated resin by Tris and incubated with a MacA-HlyD hybrid protein (D24, D18, or D12; H). After washing, the resin was applied to the SDS-PAGE gel. Only the MacA-HlyD12 hybrid protein was bound to the MacA-TolCα-coupled resin. The *E. coli* MacA (M), which was known to bind to the MacA-TolCα hybrid protein [22], was used as a positive control. B. Interaction of the *E. coli* MacA-HlyD hybrid (wild-type and mutants) and MacA-TolCα hybrid on a size-exclusion chromatographic column. Elution profiles of the wild-type MacA-HlyD12 hybrid and its mutants (R186A, L190A, T197A, and T197Y) were co-injected with the MacA-TolCα hybrid protein. The arrows with the molecular mass indicate the fractions corresponding to the calculated molecular size from the elution volume. The box around 560 kDa as a molecular size indicates complex formation from the two proteins. The elution profiles of MacA-HlyD12 hybrid proteins alone are shown in Figure S1. C. *In vitro* binding assay to confirm the results of the size-exclusion chromatography. The same proteins were used as in (B), and the experimental methods were used as in (A). Results were similar to those produced in (B), except for the augmented affinity between the MacA-HlyD12 hybrid T142A mutant and the MacA-TolCα hybrid.

To confirm whether the RLT motif of HlyD forms a binding interface with the TolC α-barrel tip region, we carried out the chimeric approach that was used in a previous study of the interactions between MacA and TolC [22]. In this study, three chimeric MacA proteins containing the RLT motif were generated. The α-hairpin tip 24 (TYSRQQRLAQTKAVSQQDLDTAAT; the underlined region indicates the RLS or RLT motif), 18 (TYSRQQRLAQTKAVSQQD), or 12 residues (RQQRLAQTKAVS) of *E. coli* MacA were substituted with 24 (DKTRLTSLIKEQFSTWQNQKYQKE), 18 (DKTRLTSLIKEQFSTWQNQKY), or 12 residues (RLTSLIKEQFST) of *E. coli* HlyD, respectively, resulting in the chimeric proteins MacA-HlyD24 hybrid, MacA-HlyD18 hybrid, and MacA-HlyD12 hybrid. To examine whether the TolC α-barrel tip region is involved in the HlyD binding, a TolC chimeric protein that contains a TolC α-barrel tip of 24 amino acids (MacA-TolCα hybrid-dimer) was used, which was previously used to show that the chimeric protein specifically binds to MacA and AcrA, and reflects the functional binding of TolC to the cognate adaptor proteins [22], [23]. With the chimeric proteins, we performed an *in vitro* binding assay on CNBr-activated agarose resin. The TolC chimeric protein (MacA-TolCα hybrid dimer) was coupled to the resin and then each MacA-HlyD hybrid protein was incubated to allow binding. As shown in Fig. 4A, only the MacA-HlyD12 hybrid was bound to the TolC α-barrel tip region. This result indicates that the 12 residues of the RLT motif comprise the binding interface with the TolC α-barrel tip, and that the conformation of the RLT is similar to that of the RLS motif in MacA (see Discussion).

To assess the importance of the conserved residues in the RLT motif, we introduced mutations to the MacA-HlyD12 hybrid and subsequently measured the affinity to the TolC chimeric protein. The TolC chimeric protein and the HlyD chimeric proteins were applied to size-exclusion chromatography to measure the binding affinity. Unlike the wild-type MacA-HlyD12 hybrid protein, the non-hemolytic *E. coli* HlyD mutant hybrid proteins containing a single amino acid substitution (R186A, L190A, T197Y) did not interact with the TolC hybrid, while the HlyD T197A variant protein showed a partial affinity for the TolC hybrid (Fig. 4B). A similar result was produced by another *in vitro* binding assay based on the TolC chimeric protein-coupled agarose resin, although the T142A variant protein showed as strong an affinity for the TolC hybrid as the wild-type HlyD hybrid protein (Fig. 4C). Combined with *in vivo* binding assays, the results of the *in vitro* binding assays showed that the HlyD RLT motif is not only functionally related to, but also interacts with, the TolC α-barrel tip region.

## Discussion

In this study, we identified the RLT motif in HlyD as a functional counterpart of the RLS motif in drug efflux pumps and demonstrated that the RLT motif of HlyD plays an important role in the action of the HlyBD-TolC translocator. A single substitution mutation in the RLT motif of HlyD abolished binding to TolC in the *in vivo* cross-linking assay and secretion of HlyA to the extracellular environment *in vivo*. A chimeric protein containing the intact RLT motif was able to bind to the TolC α-barrel-tip-containing protein, indicating that the RLT motif comprises the binding interface to the TolC α-barrel tip. Therefore, our findings imply that HlyD and TolC share the same binding mode with MacA (or AcrA) and TolC.

What does the structure of the binding domain of HlyD resemble? Many crystal structures of adaptor proteins in tripartite efflux pumps have a long, α-hairpin domain that is responsible for the TolC binding. However, the sequence alignment does not clearly define the corresponding domain in HlyD except for the RLS motif, suggesting that HlyD does not have the canonical α-hairpin domain. Furthermore, the chimeric MacA proteins harboring the HlyD 24 or 18 residues did not function as MacA surrogates in binding to the TolC chimeric protein in our results, unlike the chimeric protein with the only HlyD RLT motif. This observation suggests that the adjacent residues of the RLT motif might interfere to form the α-hairpin structure of MacA. The trimeric model of HlyD was proposed based on chemical cross-linking results [9]. However, recent studies have also converged on the hexameric model of adaptor proteins in tripartite efflux pumps, although the debate continues [11], [22], [23], [25], [29]. Therefore, a hexameric model of HlyD should be considered. The hexameric HlyD model is geometrically reasonable, because the dimeric HlyB ABC transporter and the trimeric TolC are connected by HlyD with an oligomerization number of ‘6,’ the least common multiple between 2 and 3. As supportive evidence for the hexameric model, the RLT motif of HlyD loaded into the hexameric platform of MacA showed a strong affinity for the TolC α-barrel in the binding assay using the chimeric HlyD proteins. This result further suggests that the assembly of the Hly translocator complex is similar to those of the drug efflux pumps with a modified α-hairpin domain, such that HlyD makes a direct contact with TolC through a structural motif similar to the adaptor proteins in the drug efflux pumps.

To date, the assembly of the type I secretion system remains unclear. This study reveals a crucial motif in the adaptor protein for TolC binding, and shows parallels with the assembly of tripartite efflux pumps. Although further structural and functional studies are required to envisage the fully assembled complex, this study provides insight into the assembly mechanism for a tunnel across the periplasm and through the outer membrane for toxin translocation.

## Materials and Methods

### Bacterial strains and expression plasmids for genetic studies

The non-pathogenic *E. coli* strain SE5000 (*rpsL ara139* Δ[*lacIPOZYA*]*U169 recA57 thi*) was used for the *in vivo* study. Cultures were grown at 37°C in LB medium, normally supplemented with 10 mM CaCl_2_, for the hemolytic assay. Plasmid pLG815 carried *hlyBD* genes cloned from the wild-type pathogenic strain LE2001 [30]. Plasmid pLG813 was a pACYC derivative encoding the *hlyCA* genes [31]. Plasmids pLG815 HlyD-null, HlyD-R186A, HlyD-L190A, HlyD-T197A, and HlyD-T197Y were constructed using the overlap extension PCR method. The resulting PCR product was digested with *Acc*I and *Apa*I, and ligated into the same sites in pLG815 to produce the plasmids described above. pLG815 HlyD C-*myc*, HlyD-R186A C-*myc*, HlyD-L190A C-*myc*, HlyD-T197A C-*myc*, and HlyD-T197Y C-*myc* plasmids were constructed by cloning the DNA fragment encoding the C-terminal region of HlyD with a PCR-amplified c-Myc-tag into the *Acc*I and *Xba*I sites of pLG815. Plasmid pTolC2 was constructed by subcloning the *Not*I and *Xba*I fragments containing the coding region of TolC from pTolC1 [20] into the same sites in pPM30. Primers used for plasmid generation are listed in Table S1.

### Construction of *Actinobacillus actinomycetemcomitans* (Aa) MacA-TolCα hybrid-dimer

Construction of the Aa MacA-TolCα hybrid-dimer has been previously described [22].

### Construction and expression of *E. coli* MacA-HlyD12 hybrid, *E. coli* MacA-HlyD18 hybrid, and *E. coli* MacA-HlyD24 hybrid

To construct the expression vector for the *E. coli* MacA-HlyD12 hybrid, the three DNA fragments encoding *E. coli* MacA residues 32–130, HlyD residues 186–197, and MacA residues 143–371, respectively, were joined using the overlapping PCR technique, and then ligated into the *Nco*I and *Xho*I sites of pPROEX-HTA (Invitrogen). A similar approach was utilized for construction of the expression vector for the *E. coli* MacA-HlyD18 and *E. coli* MacA-HlyD24 hybrids. For the MacA-HlyD18 hybrid, the DNA fragments encoding MacA residues 32–127, HlyD residues 183–203, and MacA residues 149–371 were joined. For the MacA-HlyD24 hybrid, the DNA fragments encoding MacA residues 32–127, HlyD residues 183–206, and MacA residues 152–371 were joined. The sequences of the primers used for the plasmids are described in Table S1. Expression and purification of the hybrid proteins were performed using the same procedure previously reported [22].

### Size exclusion chromatography

Size exclusion chromatography was performed at a flow rate of 0.5 ml/min on Superdex S-200 HR 10/30 (GE-Healthcare) that was equilibrated with 20 mM Tris buffer (pH 8.0) containing 150 mM NaCl. Before injection onto the column, 1 mg/ml of MacA-TolCα hybrid-dimer protein was incubated with the same concentration of MacA-HlyD12 hybrid or its mutant proteins (R186A, L190A, T197Y, and T197A) for 30 min at 4°C, respectively. The fractions from the column were applied to SDS-PAGE, and the gel was stained with Coomassie blue.

### *In vitro* binding assay using CNBr-activated resin

The MacA-TolCα hybrid-dimer protein protein (100 µl; 1 mg/ml) was coupled to CNBr-activated agarose resin (50 µg; GE-Healthcare) according to the manufacturer's instructions. The same amount of resin was inactivated with 100 mM Tris buffer for control. Then, 1 mg/ml of MacA or MacA-HlyD hybrid protein (D24, D18, or D12), or MacA-HlyD12 variants (wild type, R186A, L190A, T197Y, and T197A) was incubated with the protein-coupled or inactivated resin. After incubation, the resin was thoroughly washed with PBS and then subjected to SDS-PAGE for analysis. The protein bands were visualized by staining with Coomassie Blue.

### Detection of HlyA

Ten milliliters of culture was harvested by centrifugation at 6,000× g for 10 min, and then TCA (trichloroacetic acid) was added to the supernatant to a final concentration of 10% (w/v) before incubation on ice for 1 hr. The supernatant protein was pelleted by centrifugation at 16,000× g for 10 min and resuspended in 50 µl of SDS sample buffer. Ten microliters of saturated Tris solution was added to bring the sample back to neutral pH. Proteins were separated on a 10% SDS-PAGE gel and subjected to Western blot analysis using polyclonal antibodies to HlyA.

### Measurement of hemolytic activity

The procedure for measuring the hemolytic activity of *E. coli* cells has been previously described [32].

### Separation of *E. coli* membranes

The procedure for preparing membrane fraction of *E. coli* cells has been previously described [33].

### *In vivo* cross-linking assay

The procedure for *in vivo* cross-linking using Dithiobis [succinimidyl propionate] (DSP) has been previously described [9]. *E. coli* BW25113Δ*acrAB* Δ*tolC210::Tn10* cells carrying pLG815 HlyD-C-*myc* or its derivatives (HlyD-R186A-C-*myc*, HlyD-L190A-C-*myc*, HlyD-T197A-C-*myc*, and HlyD-T197Y-C-*myc*) and pTolC2 were grown in LB medium to OD_600_ = 0.7 and used for cross-linking experiments. Anti-His and anti-*myc* monoclonal antibodies were used to detect TolC and HlyD with C-terminal hexahistidine (TolC) and c-Myc tags (HlyD), respectively.

## Supporting Information

Figure S1 **Elution profiles of MacA-HlyD12 hybrid proteins on a size exclusion chromatography.** The elution volumes for the peaks are shown. The same size exclusion chromatographic column was used, and the fractions for the wild type protein (wt) were analyzed by SDS-PAGE (See Fig. 4B).
(DOCX)

Table S1 **Primers used in this study.**
(DOCX)
