# Correction: Membrane Fusion Proteins of Type I Secretion System and Tripartite Efflux Pumps Share a Binding Motif for TolC in Gram-Negative Bacteria

**DOI:** 10.1371/annotation/4d57bc21-d540-496b-8220-2e90d3586b16

**Published:** 2012-10-05

**Authors:** Minho Lee, So-Young Jun, Bo-Young Yoon, Saemee Song, Kangseok Lee, Nam-Chul Ha

There were errors in the author affiliations. The affiliations should appear as shown here:

Minho Lee^1#^, So-Young Jun^2#^, Bo-Young Yoon^2^, Saemee Song^1^, Kangseok Lee^1*^, Nam-Chul Ha^2*^

**1** Department of Life Science, Chung-Ang University, Seoul, Republic of Korea, **2** Department of Manufacturing Pharmacy, College of Pharmacy, Research Institute for Drug Development, Pusan National University, Busan, Republic of Korea 

