# Supplementary material for: Membrane Fusion Proteins of Type I Secretion System and Tripartite Efflux Pumps Share a Binding Motif for TolC in Gram-Negative Bacteria
Source: PLoS One. 2012 Jul 6;7(7):e40460. doi: 10.1371/journal.pone.0040460 (PMC3391258; doi:10.1371/journal.pone.0040460)

**Figure S1. Elution profiles of MacA-HlyD12 hybrid proteins on a size exclusion chromatography.** The elution volumes for the peaks are shown. The same size exclusion chromatographic column was used, and the fractions for the wild type protein (wt) were analyzed by SDS-PAGE (See Fig. 4B)


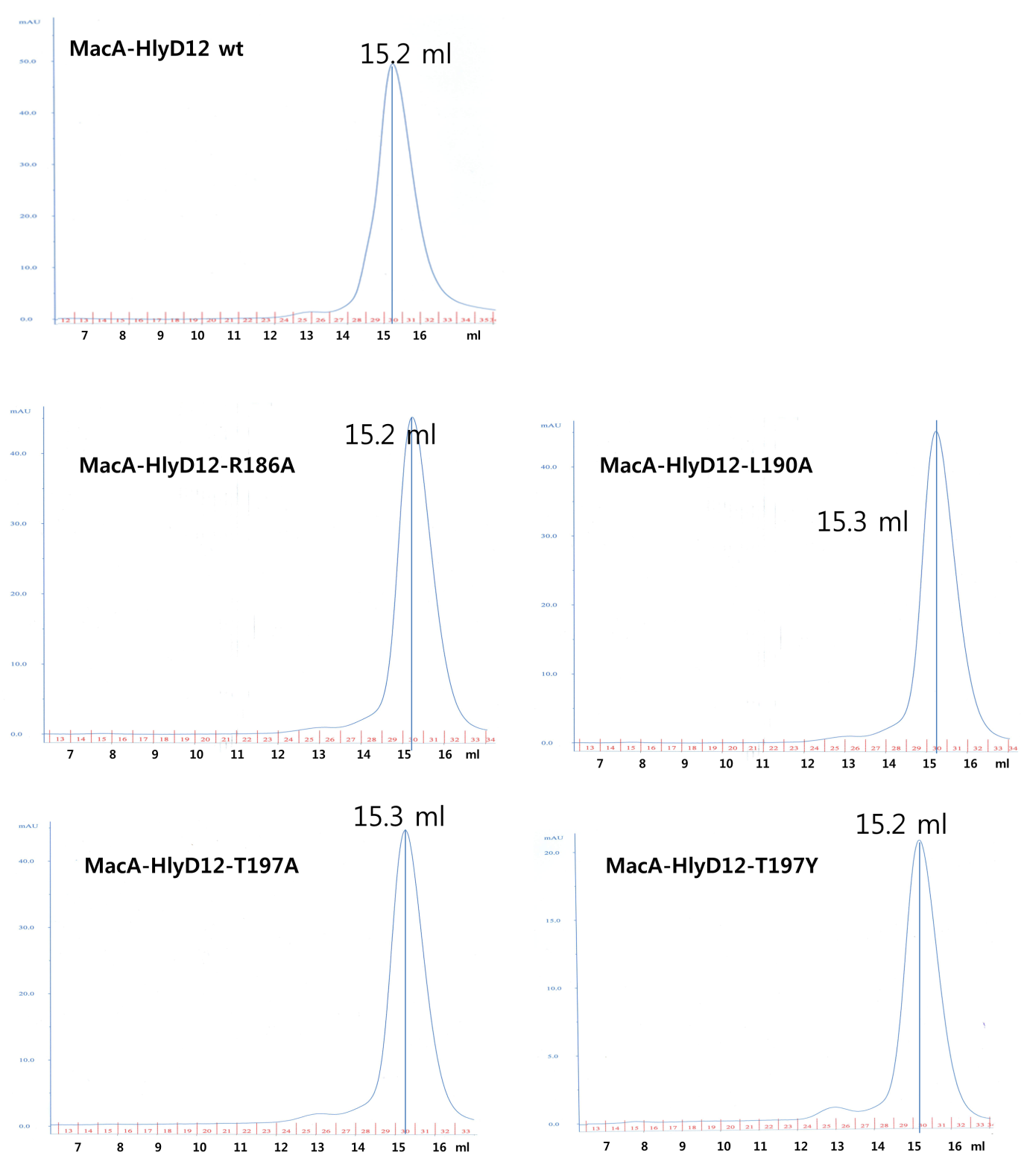

Supplement: Figure S1 — Elution profiles of MacA-HlyD12 hybrid proteins on a size exclusion chromatography. The elution volumes for the peaks are shown. The same size exclusion chromatographic column was used, and the fractions for the wild type protein (wt) were analyzed by SDS-PAGE (See Fig. 4B). (DOCX) [file pone.0040460.s001.docx]
