# Supplementary material for: Membrane Fusion Proteins of Type I Secretion System and Tripartite Efflux Pumps Share a Binding Motif for TolC in Gram-Negative Bacteria
Source: PLoS One. 2012 Jul 6;7(7):e40460. doi: 10.1371/journal.pone.0040460 (PMC3391258; doi:10.1371/journal.pone.0040460)
Supplement: Table S1 — Primers used in this study. (DOCX) [file pone.0040460.s002.docx]

**Table S1. Primers used in this study**

| **Plasmids** | **Cloning sites** | **Primers (5’ to 3’)** |
| --- | --- | --- |
| pLG815 HlyD-stop | *Acc*I/*Apa*I | HlyB F (*Acc*I) 5’GTCTGTCTACAGTAAAAAAT |
|  |  | HlyD Stop R 5’ACCATGTTTTCTAATTCTTC |
|  |  | HlyD Stop F 5’GAAGAATTAGAAAACATGGT |
|  |  | HlyD R (*Apa*I) 5’AACAGGGGCCCTGATTACTG |
| pLG815 HlyD-R131A | *Acc*I/*Apa*I | HlyB F (*Acc*I) 5’GTCTGTCTACAGTAAAAAAT |
|  |  | HlyD R131A R 5’GAAGTTAAAGCCAGTACTTC |
|  |  | HlyD R131A F 5’GAAGTACTGGCTTTAACTTC |
|  |  | HlyD R (*Apa*I) 5’AACAGGGGCCCTGATTACTG |
| pLG815 HlyD-L135A | *Acc*I/*Apa*I | HlyB F (*Acc*I) 5’GTCTGTCTACAGTAAAAAAT |
|  |  | HlyD L135A R 5’TCTTTTATAGCAGAAGTTAA |
|  |  | HlyD L135A F 5’TTAACTTCTGCTATAAAAGA |
|  |  | HlyD R (*Apa*I) 5’AACAGGGGCCCTGATTACTG |
| pLG815 HlyD-T142A | *Acc*I/*Apa*I | HlyB F (*Acc*I) 5’GTCTGTCTACAGTAAAAAAT |
|  |  | HlyD T142A R 5’TTTTGCCAAGCGGAAAACTG |
|  |  | HlyD T142A F 5’CAGTTTTCCGCTTGGCAAAA |
|  |  | HlyD R (*Apa*I) 5’AACAGGGGCCCTGATTACTG |
| pLG815 HlyD-T142Y | *Acc*I/*Apa*I | HlyB F (*Acc*I) 5’GTCTGTCTACAGTAAAAAAT |
|  |  | HlyD T142Y R 5’TTTTGCCAATAGGAAAACTG |
|  |  | HlyD T142Y F 5’CAGTTTTCCTATTGGCAAAA |
|  |  | HlyD R (*Apa*I) 5’AACAGGGGCCCTGATTACTG |
| pLG815 HlyD C-*myc* | *Acc*I/*Xba*I | HlyB F (*Acc*I) 5’GTCTGTCTACAGTAAAAAAT |
|  |  | HlyD-myc R (*Xba*I)  5’AATCTAGATTACAGATCCTCTTCTGAGATGAGTTTTTGTTCACGCTCATGTAAACTTTCTGTTAC |
| pLG815 HlyD R131A C-*myc* | *Acc*I/*Xba*I | HlyB F (*Acc*I) 5’GTCTGTCTACAGTAAAAAAT |
|  |  | HlyD-myc R (*Xba*I)  5’AATCTAGATTACAGATCCTCTTCTGAGATGAGTTTTTGTTCACGCTCATGTAAACTTTCTGTTAC |
| pLG815 HlyD L135A C-*myc* | *Acc*I/*Xba*I | HlyB F (*Acc*I) 5’GTCTGTCTACAGTAAAAAAT |
|  |  | HlyD-myc R (*Xba*I)  5’AATCTAGATTACAGATCCTCTTCTGAGATGAGTTTTTGTTCACGCTCATGTAAACTTTCTGTTAC |
| pLG815 HlyD T142A C-*myc* | *Acc*I/*Xba*I | HlyB F (*Acc*I) 5’GTCTGTCTACAGTAAAAAAT |
|  |  | HlyD-myc R (*Xba*I)  5’AATCTAGATTACAGATCCTCTTCTGAGATGAGTTTTTGTTCACGCTCATGTAAACTTTCTGTTAC |
| pLG815 HlyD T142Y C-*myc* | *Acc*I/*Xba*I | HlyB F (*Acc*I) 5’GTCTGTCTACAGTAAAAAAT |
|  |  | HlyD-myc R (*Xba*I)  5’AATCTAGATTACAGATCCTCTTCTGAGATGAGTTTTTGTTCACGCTCATGTAAACTTTCTGTTAC |
| pTolC2 | *Not*I/*Xba*I | pKAN6B forward2 5’TTCTGTAACAAAGCGGGACC |
|  |  | tolC-R (*Xba*I)  5’ GGGTCTAGATCAATGATGATGATGATGATGGTTACGGAAAGGGTT |
